# Detection and Selective Sorption of Copper Ions by a COF-Modified Melamine Sponge

**DOI:** 10.1021/acsomega.5c01393

**Published:** 2025-05-21

**Authors:** Panagiota Bika, Nikolaos Ioannidis, Polychronis Tsipas, Stefanos Papagiannis, Maria-Anna Gatou, Evangelia A. Pavlatou, Andreas Germanos Karydas, Thomas Stergiopoulos, Panagiotis Dallas

**Affiliations:** † Institute of Nanoscience and Nanotechnology, 54572 NCSR Demokritos, 15341 Athens, Greece; ‡ Institute of Nuclear and Particle Physics, NCSR Demokritos, 15341 Athens, Greece; § National Institute of Materials Physics, Atomistilor 405A, Magurele 077125, Romania; ∥ Laboratory of General Chemistry, School of Chemical Engineering, 68994 National Technical University of Athens, Zografou Campus, 15772 Athens, Greece; ⊥ Theoretical and Physical Chemistry Institute, National Hellenic Research Foundation, 11635 Athens, Greece

## Abstract

Commercial melamine sponges were modified with a functional covalent organic framework (COF), and they were evaluated as adsorbents of divalent copper cations from aqueous solutions. A phosphazene unit successfully covered the surface of the melamine sponge, and the organic framework was subsequently formed through the nucleophilic substitution with 4,4′ bipyridine. The covalent organic framework functionalized on the melamine sponge can detect and effectively adsorb copper compounds in aqueous solutions. Its selectivity toward the adsorption of copper was demonstrated through the presence of different metal salts. Four competitive metal cations, i.e., copper, nickel, iron, and calcium, were selected to confirm the preferential binding of copper on the COF-functionalized sponge. The outcome was determined through the studies of X-Ray Fluorescence elemental analysis, X-Ray Photoelectron Spectroscopy (XPS), and Electron Paramagnetic Resonance experiments. XRF reported a copper sorption capacity of 293 μg cm^–2^, which is nearly nine times higher than the performance of the pristine sponge. Q-band EPR measurements demonstrated the presence of different coordination sites with different substituents for copper on the modified sponges, when the adsorption took place in an aqueous solution containing exclusively copper cations, while only one coordination, the favorable trigonal bipyramidal geometry, was obtained in the presence of additional metals.

## Graphical abstract

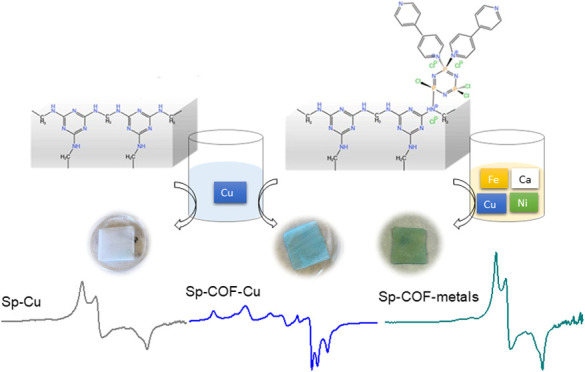

## 1 Introduction

The ongoing development of industrialization and the excessive usage of transition and radioactive metals have generated immense environmental pollution around the globe, disrupting the natural balance of the aquatic, soil, and atmospheric environment. Toxic heavy metals,^1^ nuclear waste,^2^ and organic pollutants are increasingly accumulating in the biological systems,^3^ eroding in the aquatic environment and the soil, participating in the food chain and threatening the life of living organisms.

Wastewater discharge from mines and industries has been the primary source of water contamination.^4^ Unlike organic pollutants,^3^ metals cannot be easily degraded into eco-friendly substances. Traditional techniques for the extraction and recovery of heavy metals have been established and include chemical precipitation, sorption, solvent extraction, ion exchange, electrolysis, filtration, and photocatalytic reduction. All of them are thoroughly presented in a comprehensive review by Qasem et al.^5^ Since pollutant sources contain different metals, a more selective sorption-based extraction was encountered with the use of adsorbent materials, such as silica gel and clays,^6^ biochar,^7^ organic resins,^8^ activated carbon,^9^ carbon nanotubes,^10^ graphene oxide, and metal–organic frameworks (MOFs).^11^

Covalent Organic Frameworks (COFs) are a unique and versatile class of advanced functional materials that have found applications in photocatalysis,^12^ heavy metal absorption,^13^ as well as passivating agents in perovskite photovoltaics.^14^ Compared to their inorganic counterparts, the metal–organic frameworks, COFs, present advantages, such as abundant binding sites, porosity with regular channels, and lower toxicity.^15^ Nowadays, environmental technologies urgently require the incorporation of eco-friendly materials with high surface areas, chemical and thermal stability, while being prone to recyclability and regeneration. Additionally, their costless and easy synthesis is necessary for attainable scale-up production. The multifunctionality provided by the COFs for the detection, determination, and sorption of heavy metals is utilized for the capture of the toxic ions from the contaminated water hastily and efficiently. Furthermore, specific functional groups^16^ of the COFs can further improve the selectivity of the metals. Their adsorption takes place through ion exchange,^17^ electrostatic attraction,^18^ hydrogen bonding,^19^ coordination bonds,^20^ and chelation,^21^ with the latter two offering the most stable immobilization. COFs as advanced absorbents were also combined with melamine sponges in order to prepare virtuosity composite materials of multifunctionality for a wide application scope.However, there are some drawbacks since the uptake and the final performance of the materials vary with the pH, temperature, competitive metals, and coexisting substances in the solution.^22^

Recently, we demonstrated the ultraefficient removal of copper from an aqueous solution by utilizing two different frameworks, based on either a phosphazene or a triazine core.^23^ With respect to our previous work, we employed commercial melamine sponges as a support for the functionalization of a covalent organic framework, while its design targets a high adsorption efficiency of heavy metals from aqueous solutions. Control experiments were first carried out with the pristine sponge, as the reference scaffold, for the removal of copper, nickel, iron, and calcium. Afterward, the two systems, the pristine and the COF-modified sponge, were studied and compared for their adsorption capacity to remove copper and other competitive metals from aqueous solutions. The experiments and the detailed characterization of their results demonstrated that only the modified melamine sponge with the COF efficiently subtracts the divalent copper cations from the aqueous solution. The recuperation of the formed complexes with the functionalized COF on the sponge occurs at ease, and complete demetallization takes place by adding a dilute acidic solution. Its removal ability of copper ions surpasses that of commercial sponges, even in the presence of additional competitive metal ions. This first modified sponge for the selective removal of copper from aqueous solutions is thoroughly evaluated for its advantages.

## 2 Experimental Procedure

### 2.1 Materials

Commercial melamine sponges, phosphonitrilic chloride trimer (N_2_P_3_Cl_6_) 99%, and toluene 99.5% (C_6_H_5_CH_3_) were purchased from Sigma-Aldrich, and 4,4′ bipyridine 98% (C_10_H_8_N_2_) was purchased from Thermo Scientific. As for the metals used, copper sulfate pentahydrate (CuSO4·5H2O) was purchased from panreac quimica SA, nickel sulfate tetrahydrate >90% (NiSO_4_·4H_2_O) was purchased from Fluka, calcium nitrate tetrahydrate 99+% (Ca­(NO_3_)_2_·4H_2_O) was purchased from Chem lab NV and iron nitrate nonahydrate 98+% (Fe­(NO_3_)_3_·9H_2_O) was purchased form ChemLAB NV.

### 2.2 Synthesis and Functionalization of COF on Modified Sponge

The in-situ synthesis of the foreseeable COF on the melamine sponge was performed in a two-step, bottom-up procedure at room temperature without requiring any harsh conditions. Commercial melamine sponges cut into (2 cm × 2 cm × 0.5 mm) dimensions were embedded in a 30 mL toluene solution containing 750 mg of phosphonitrilic chloride trimer (P_3_N_3_Cl_6_). The sponge was left to react for 24 h with mild stirring. The modified sponge with P_3_N_3_Cl_6_ was then washed with toluene to remove the excessive precursor, and it was then embedded into a solution of 450 mg 4,4′ bipyridine in 30 mL of toluene for 24 h under mild stirring. The final product of the modified sponge with the COF was washed with toluene to remove any unreacted precursor. The pristine sponge is referred to with the abbreviation Sp and the modified sponge with the abbreviation Sp-COF.

### 2.3 Adsorption Experiments

The modified sponges with the COF were impregnated into a solution of CuSO_4_·5H_2_O (4 mg/mL) and were left static for 24 h to ensure a good diffusion and a complete adsorption until saturation of their sites (sample denoted as Sp-COF-Cu). The same procedure was followed for the pristine melamine sponge (sample denoted as Sp-Cu). In the end, both sponges were cleansed with distilled water to remove any physisorbed quantities. The pristine and modified sponges were impregnated in a second solution, containing 1000 ppm of each inorganic compound, followed by: Ca­(NO_3_)_2_·4H_2_O, Fe­(NO_3_)_3_·9H_2_O, CuSO_4_·5H_2_O, and NiSO_4_·4H_2_O. The system was left static for 24 h, and then, both sponges after the adsorption, were cleansed with water to extract the physisorbed quantities of the metals. Correspondingly, the samples are denoted as Sp-COF-metals and Sp-metals.

## 3 Characterization Techniques

The surface morphologies were examined with Scanning Electron Microscopy (SEM) using a JEOL 7401f Field Emission. The crystalline structure was analyzed by using X-ray diffraction (XRD) patterns from 2 to 80° 2θ, which were obtained with a Smart Lab Rigaku diffractometer (Cu Ka radiation). FTIR spectra were recorded on a Thermo Nicolet iS50 instrument in attenuated total reflection mode from 400 to 4000 cm^–1^. X-ray photoelectron spectroscopy (XPS) was carried out to analyze the chemical state and composition of the pristine and modified sponges before and after functionalization and the metals’ adsorption. The XPS measurements were performed using a Mg Ka X-ray source with a photon energy of 1253.64 eV, and the spectra were collected with a PHOIBOS 100 (SPECS) hemispherical analyzer. Gaussian–Lorentzian shapes (Voigt functions) were used for fitting of the recorded spectra after standard Shirley background subtraction. Furthermore, to trace both minor and major elements, the samples were analyzed using a high-resolution energy-dispersive X-ray fluorescence (ED-XRF) 3D optics spectrometer, Epsilon 5 (PANalytical). This spectrometer features an X-ray tube with a W/Sc anode positioned with a side-window configuration. The X-ray radiation emitted by the sample is detected by a liquid-nitrogen-cooled Ge detector, offering an energy resolution of approximately 150 eV fwhm at Mn–Kα (5.89 eV). For optimal analysis, six secondary targets (CaF_2_, Ge, Mo, KBr, LaB_6_, and Al_2_O_3_) were used to both polarize the primary X-ray tube radiation and generate a high-intensity, bichromatic beam from the K-fluorescence lines of each secondary target. All measurements were conducted under vacuum, and the total analysis time per sample was about 60 min. The methodology for the elemental characterization is explained in detail in previous works;^24^ the measured samples were considered infinitely thin, and the concept of elemental sensitivities obtained experimentally from certified thin sample materials was used for quantification purposes. Additionally, to ensure accuracy, blank subtractions were performed on the pristine sponge and modified sponge with the COF before the analysis of the elements after the metals’ adsorption. The concentration and limits of detection (LOD) are provided in μg cm^–2^ and the uncertainty in % for each element (Sp-Cu, Sp-COF-Cu, Sp-metals, and Sp-COF-metals). Moreover, dynamic adsorption experiments were carried out using an experimental setup of three small-scale columns, in order to examine the reproducibility of the results. The prepared columns were adjusted to three-channel microflow variable-speed peristaltic pumps, enabling the simultaneous upward inlet of the solutions into 3 columns. The columns were washed for 24 h with double-deionized water prior to the adsorption experiments. After the beds’ (samples) saturation, the copper aqueous solution was injected through the columns with an upward flow of 0.35 mL/min at ambient temperature. The Cu adsorption efficiency of the sponges was evaluated with an initial Cu concentration of ∼10 mg L^–1^. The inlet concentration of the Cu­(II) solution is denoted as *C*_0_, mg L^–1^, and the final effluent is denoted as *C_t_*, mg L^–1^ in relation to time (*t*, min), and both are represented by the average value of the three replicate columns at the same time interval. The standard deviation is represented by the relative difference of the measurements, and the efficiency is calculated by (*C*_0_ – *C_t_*)/*C*_0_. The effluents, collected from the top of the columns, were stored at 4 °C prior to analysis by Flame Atomic Absorption Spectroscopy, F-AAS, (PinAAcle 500 series, PerkinElmer), calibrated with 2.5, 5.0, and 10.0 mg L^–1^ standard solutions (Sigma-Aldrich). The sponges were afterward cut into smaller pieces to conduct the Electron Paramagnetic Resonance. EPR measurements at the Q-band were performed on a home-assembled spectrometer equipped with an ER 5106 QT Bruker resonator, an Anritsu MF2414C microwave frequency counter, and a CF935P Oxford Instruments helium cryostat. The temperature was controlled using an Oxford ITC 4 temperature controller. Spectra of Sp-COF-Cu and Sp-Cu were collected at room temperature, while those of Sp-COF-metals at 28 K with the following conditions: microwave frequency, 34.04 GHz; microwave power, 0.41 mW; modulation frequency, 100 kHz; modulation amplitude, 0.1 mT. EPR spectra were analyzed and simulated using the EasySpin package.^25^

## 4 Results and Discussion

### 4.1 Copper Adsorption

In Figure 1, we present the steps followed for the modification of the melamine sponge with functional covalent organic frameworks. The modification procedure involves aSchiff-base reaction of phosphonitrilic chloride with the free nitrogen of the melamine backbone (step 1) and, afterward, the nucleophilic substitution of the unreacted chloride sites by the electron pair of nitrogen of 4,4′ bipyridine units (step 2). The modification did not induce any visible color changes, since the synthesized and interpenetrated COF is white. Once the pristine and modified sponges were immersed in an aqueous copper sulfate solution, an immediate change of color from white to cyan blue (Figure 1b) was observed for the modified Sp-COF, indicating its quick complexation with copper. This instantaneous complexation of the COF with copper was previously encountered by Bika et al.^23^ In contrast, the pristine sponge that was also impregnated into the same divalent copper solution did not demonstrate the same visual response, with only a very minor percentage of colored areas observed.

**Figure 1:**
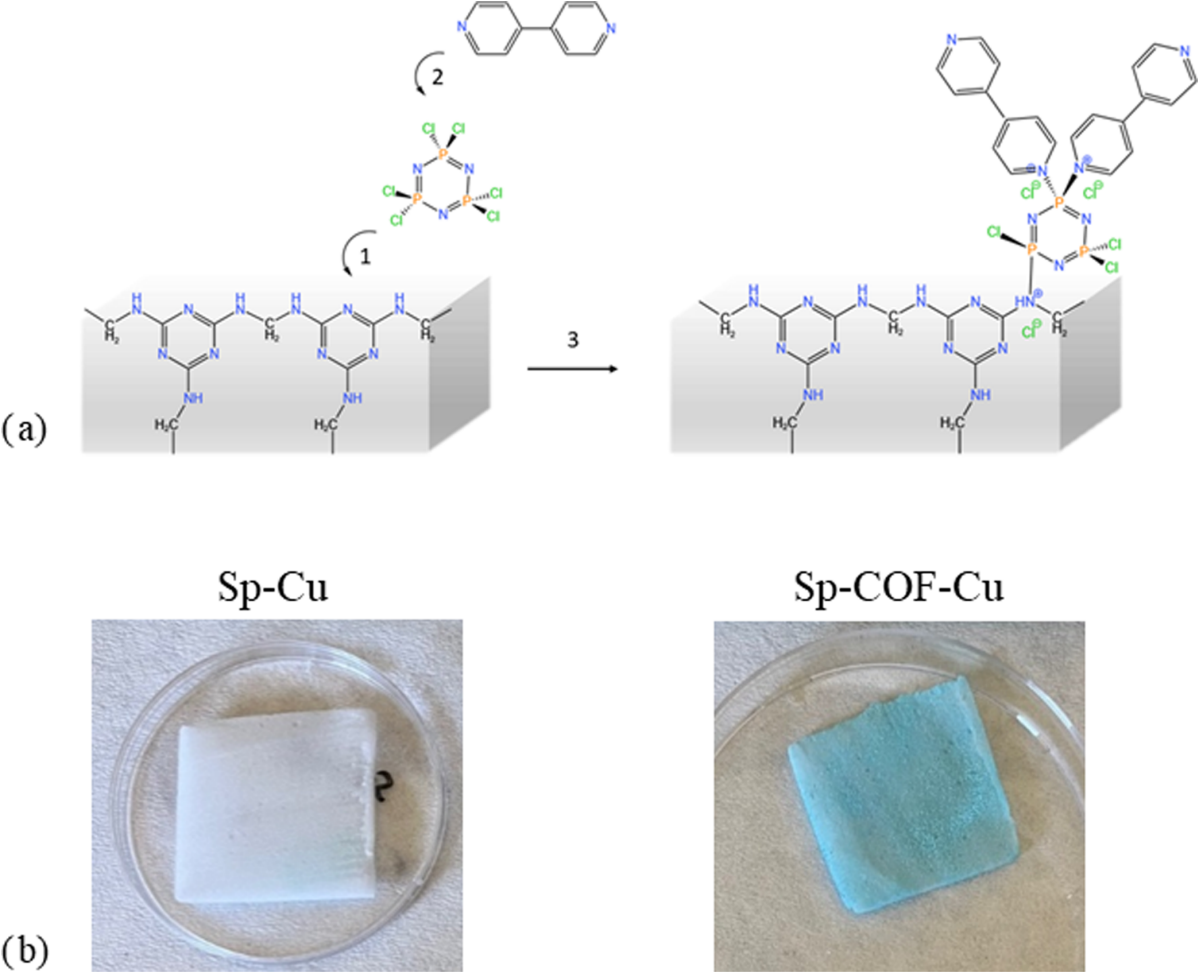
(a) Steps for the modification of the melamine sponge with the COF: (1) functionalization of phosphazene core, (2) attachment of 4,4′ bipyridine, and (3) the final-modified Sp-COF. (b) Images of the pristine and the modified sponge after absorption of copper cations.

The successful functionalization of the melamine sponges was initially demonstrated by FTIR spectroscopy. The commercial sponges consist of a formaldehyde-melamine-sodium bisulfite copolymer, and their functional groups are easily detected with vibrational spectroscopy tools. In Figure S1a, the FTIR spectrum of the pristine Sp presents its characteristic symmetric stretching vibrations of CH_2_ at 2951, 2917, and 2848 cm^–1^, in consistency with other studies.^26^ The vibrations of the secondary amine at 3400 cm^–1^ are not evident but lie underneath the broad adsorption peak of absorbed humidity. At the lower frequency range,^26^ the bending of the main triazine ring appears at 809 cm^–1^, along with the C–O at 1124 cm^–1^, the C–N triazine ring at 1324 cm^–1^, the C–H bending of the methyl group at 1450 cm^–1^, the C–N–H vibration at 1462 cm^–1^, and the stretching vibration of C=N located at 1544 cm^–1^. On the other hand, looking into the spectra of Sp-COF in Figure S1, new prominent vibrations manifest, and they are assigned to the molecular structure of the COF attached to the melamine sponge. The P–Cl bond of phosphonitrilic chloride trimer, with its double peaks located at 522 and 603 cm^–1^,^27^ appears with a rather weak intensity. Its presence indicates that some parts of the phosphazene ring remained unreacted due to steric hindrance and thus, a quasi-limited diffusion. However, the weak intensity of this peak proves the successful step reactions of the precursor molecules on the functional surface of the melamine. The P=N vibrations are located at 1216 cm^–1^. The spectrum of CuSO_4_·5 H_2_O was collected to identify its vibrations at the final samples, and along with the Sp, Sp-Cu, Sp-COF, and Sp-COF-Cu are all presented in Figure S1. The vibrations related to CuSO_4_·5 H_2_O are found at 606, 870, 972, 1070, and 1666 cm^–1^. Specifically, the asymmetric SO_4_^2–^ peak at 1070 cm^–1^ is present in all samples, which were treated with copper salt.^28^ While the C–N and C=N regions, 1300–1600 cm^–1^, remain practically the same in the Sp and Sp-Cu samples, pronounced changes are indicated in the Sp-COF and Sp-COF-Cu spectra. This is the first proof that the bipyridine moieties strongly coordinate with the copper cations. Specifically, peaks from the triazine ring appear at 1404, 1486, 1522, and 1597 cm^–1^, while the −C=N at 1637 cm^–1^ shifts to 1611 cm^–1^ after copper’s complexation, in accordance with previous references.^29^

Before and after chemical modification of the pristine sponge, the XRD patterns were collected to identify the alterations in the structure and crystallinity of the COF-modified sponge. The patterns of the Sp, Sp-Cu, Sp-COF, and Sp-COF-Cu samples are presented in Figure S1b. The patterns in general, possess an amorphous broad background due to the melamine sponge and its support on a glass substrate with tack. The diffractogram of the latter is included in the Supporting Information section (Figure S2), and the peaks assigned to it are indicated with red stars in the diffractograms. The new superimposed diffraction peaks of the COF rose at 10.58° (8.36 Å), 12.59° (7.03 Å), 14.03° (6.31 Å), 19.61° (4.52 Å), 24.71° (3.6 Å), 25.59° (3.48 Å), 25.88° (3.44 Å), and 26.9° (3.31 Å) confirm that the framework retains a crystallinity on the surface of the sponge. The peaks are consistent with previous studies, albeit with a slight shift.^23^ For example, the reflection at 10.58° is close to that of 4,4′ bipyridine, and the reflection at 12.59°, which is near the triazine core, could be assigned to the phosphazine core. An in-plane stacking pattern could be recognized at 14.03° if the intensity of the measurement was sharper, whereas 4,4′ bipyridine moieties reflect at 19.61 and 24.71°. The interplanar spacing of 3.4 Å is equivalent to the π–π stacking of the COFs, which is created, moreover, thanks to the predefined orientation of the melamine network. In the end, after the adsorption of divalent copper, the XRD pattern of the Sp-COF-Cu is largely the same as the Sp-COF, with a minor shift to higher *d*-spacings for the peaks at lower angles, which are indicated with green arrows. The Sp-COF-Cu presents a more crystalline structure. The complexation of the Sp-COF with copper is evident by the formation of new pores, reflecting at 8–14°, as encountered previously in ref 23.

The microstructure of the sponges was revealed by SEM images. The images are shown in Figure 2. The melamine sponge has a 3D intersectional system with a hierarchical and highly porous structure. After the functionalization of COF, the modified sponges’ 3D microstructure was preserved, while the morphology of their surface was radically roughened. The COF had been intercalated within the melamine network, ensuring a firm coating, as observed by the densely packed representations in Figure 2b. After the adsorption experiment with copper sulfate pentahydrate, the pristine and modified sponges retained their microstructure (Figure 2c,d). However, in both samples, there are evident modifications at their surfaces and their interconnected system. Subtle differences can be observed in the two samples, with the Sp-Cu presenting a more uniform surface compared to the Sp-COF-Cu, where, in the latter, densely and irregular, immense and intercalated quantities of the complexed copper on the COF prevail. The EDX mapping, along with other techniques, further validates this statement. The morphology of the thin COF representations has been replaced by circular dense aggregates, as encountered in a previous work,^23^ induced by the binding of the metal cations on preferred units.

**Figure 2:**
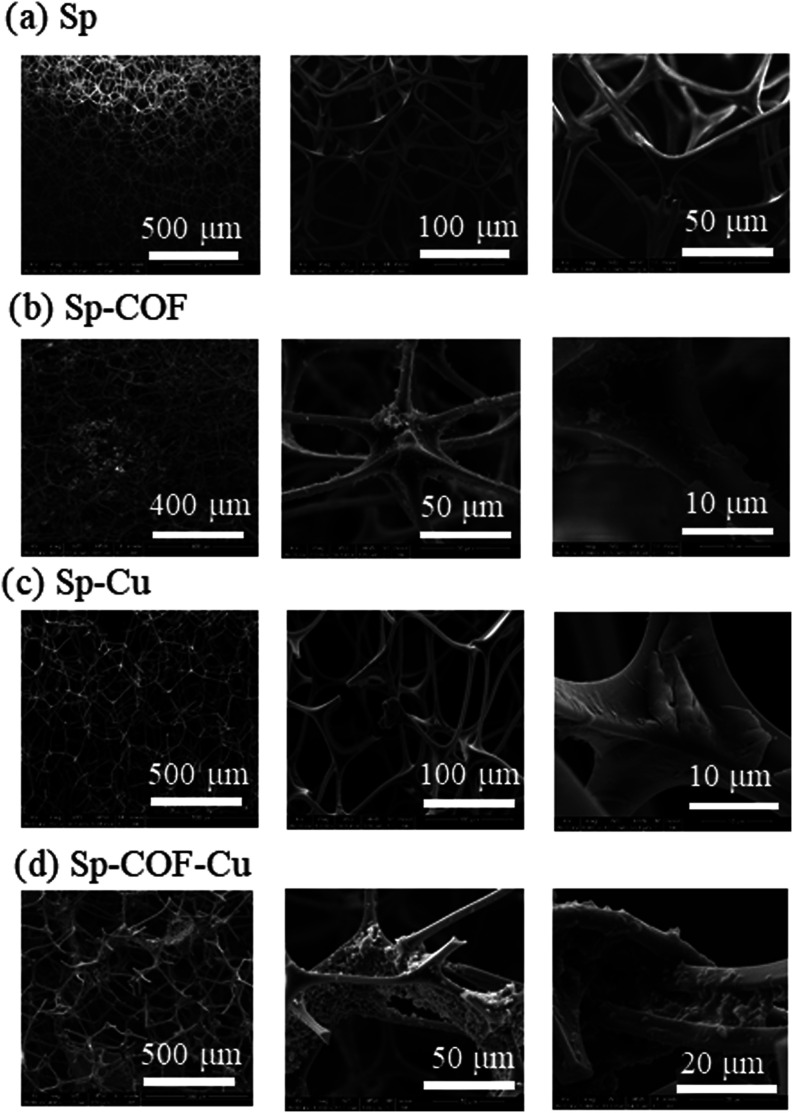
SEM images of the (a) Sp, (b) Sp-COF, (c) Sp-Cu, and (d) Sp-COF-Cu.

The EDX mapping and its spectrum in Figure 3 further verify the growth of the framework on the melamine sponge with the identification of P and Cl elements and the presence of the copper ions, coinciding in the same areas. The EDX mapping of the C and N is additionally provided in the Supporting Information (Figure S3).

**Figure 3:**
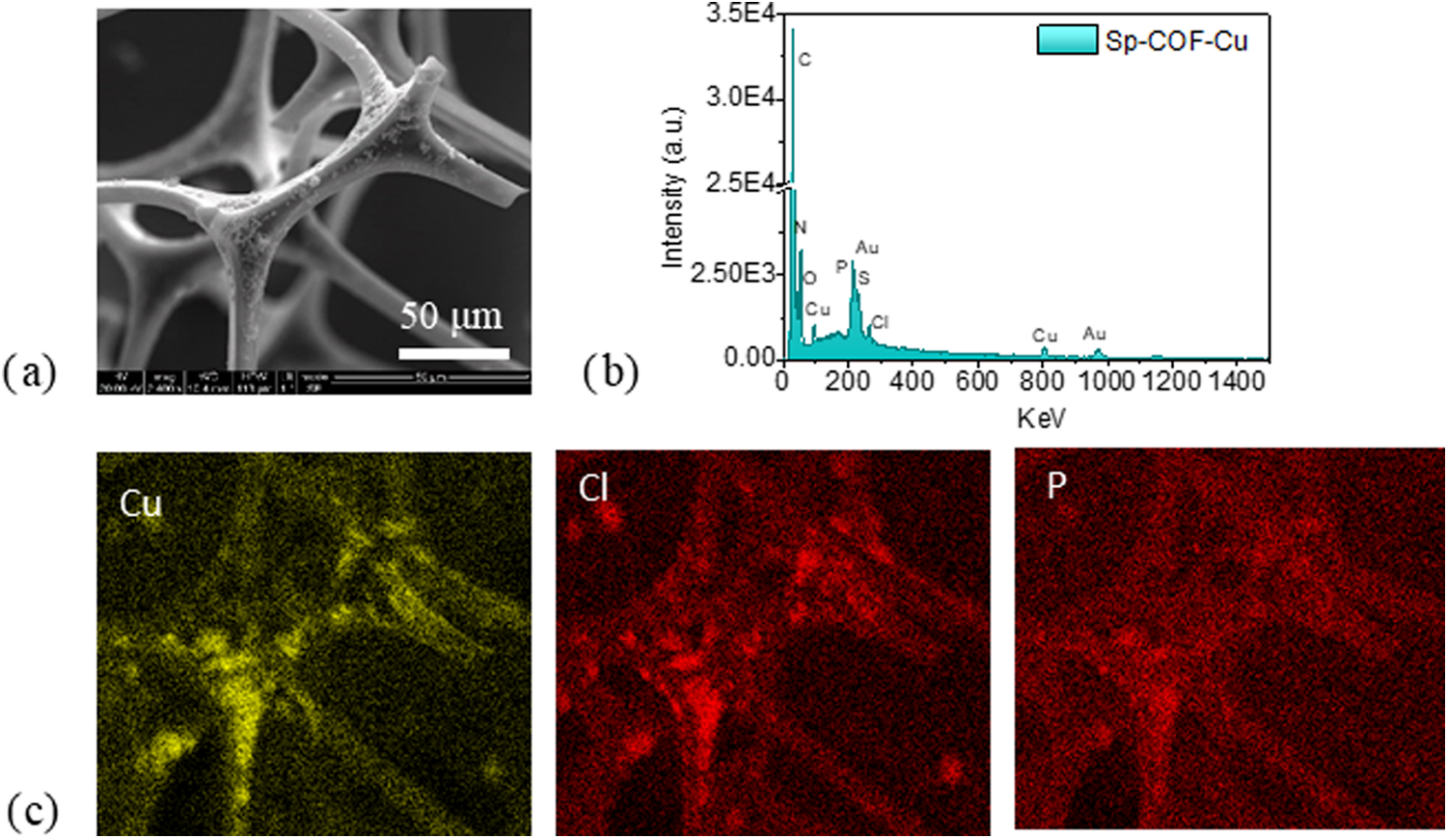
(a) SEM image of a selected region in SP-COF-Cu, (b) its EDX spectrum, and (c) its EDX mapping of the Cu, Cl, and P elements.

To identify the nature of the bonding between the attached COF to the surface of the sponge and the copper cations, an XPS study was conducted. The full survey scans, regarding the Sp-COF and Sp-COF-Cu, are included in Figure S4. The spectra and the corresponding fitting curves for the C 1s, P 2p, and N 1s regions of the Sp-COF and Sp-COF-Cu samples are demonstrated in Figure 4, while the S 2p, Cl 2p, and O 1s are included in the Supporting Information section (Figures S5 and S6). Τhe Cl 2p­(3/2) spectrum (Figure S5) presents a component with binding energy at 199.8 eV attributed to the counterbalancing chloride ions^30^ and the minor quantity of unsubstituted P–Cl, due to steric hindrance or limited diffusion. The O 1s XPS spectrum in Figure S6 features two peaks at 532.3 and 530.6–530.9 eV, highly associated with H_2_O from the surface layer and the low energy component of hydroxyl groups in the lattice, respectively.^31,32^ The C 1s XPS of the Sp-COF demonstrates two components at binding energies 284.8 and 286 eV. These are well-known to originate from −C–C–/–C=C– and −C–N bonds, respectively.^33,34^ After copper coordination, two new peaks appear at 287.7 and 288.8 eV. Since the O 1s spectra (Figure S6) of the samples before and after complexation are nearly the same, these new peaks in the carbon region cannot be assigned to the formation of −C–O or O–C=O bonds after simply the addition of copper sulfate. Thus, the peaks at BE of 287.5 and 288.8 eV are strongly indicative of a −C=N– and −C–N-metal bond,^31,34–36^ from the successful complexation on the bipyridine units. At the high-resolution P 2p­(3/2) spectrum of Sp-COF, we observe two peaks centered at 133 and 133.8 eV, both related to the phosphazene core. According to Vassileva et al.,^37^ the substitution of the chlorine from less electronegative ligands, such as pyridine units, leads to a decrease in the binding energy of the phosphorus in the central ring. Hence, we assign the peak at 133.8 eV to nonsubstituted phosphorus with 4,4′ bipyridine and the dominant peak at 133 eV to phosphorus atoms substituted with the nitrogen group. After complexation with copper cations, a significant shift to higher binding energies occurs in some elements, indicating a reduced electron density. This is expected after coordination with a more electropositive transition metal cation. The Cl 2p­(3/2) spectra (Figure S5a­(i,ii)) remained stable at 199.8 eV, as expected, due to their action as counterbalancing anions or the unreacted moieties of P–Cl, while the S 2p peak (Figure S5b) demonstrates that sulfate ions are trapped inside the COF and sponge pores. Moreover, the high-resolution N 1s spectrum of Sp-COF and Sp-COF-Cu is presented in Figure 4iii. The broad experimental band was deconvoluted into two features. The binding energies at 398.5 and 400.3 eV coincide well with the (−C=N–C) binding of pyridinic unit (C_5_H_5_N)^38^ in the triazine ring of melamine, in 4,4′ bipyridine, and the (−P=N–P) binding of phosphonitriclic chloride trimer (NPCl_2_)_3_, respectively. Τhe XPS outcome, along with the other techniques, clarifies the established functionalization of the COF on the melamine sponge. After the adsorption experiment, focusing on the N 1s region of Sp-COF-Cu, a new peak evolves, in addition to those previously reported with BE 398.5 and 400.3 eV.^31^ The binding energy of 397.8 eV is attributed to the chelated N atoms and specifically to the covalent bond of Cu–N formed successfully between N of 4,4′ bipyridine with divalent Cu.^39^

**Figure 4:**
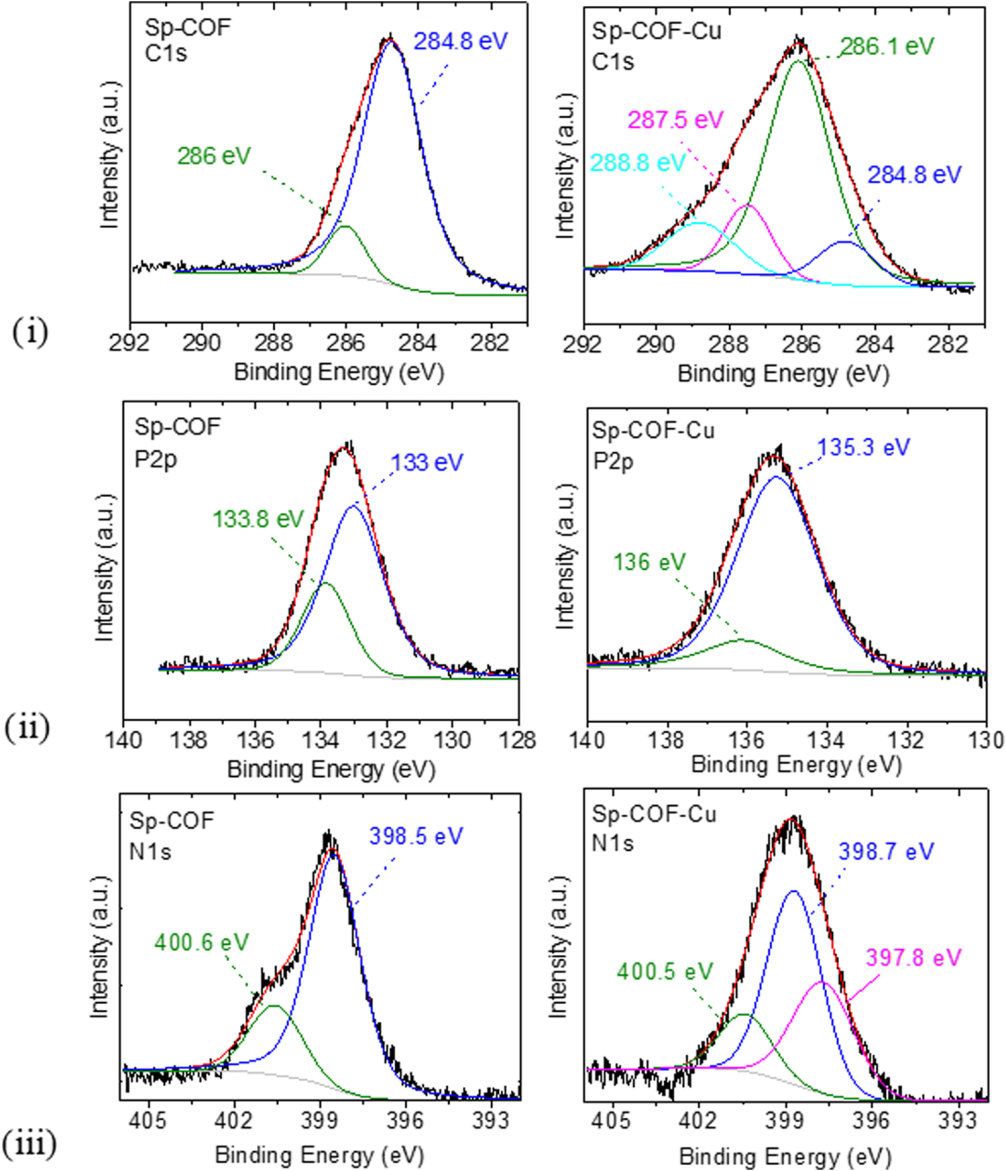
XPS spectra of the Sp-COF (left) in comparison with the Sp-COF-Cu (right) samples: (i) C 1s, (ii) P 2p, and (iii) N 1s.

The Cu 2p­(3/2) XPS spectrum of the Sp-COF-Cu samples consists of two components (Figure 5a), with their fitted peaks centered at 933 and 935.5 eV. The BE of 935.5 eV coincides well with Cu­(II) bonded with SO_4_^2–^ and monovalent anions such as Cl^–^ and NO_3_^–^. The lack of any peaks in binding energies lower than 933 eV confirms the absence of the monovalent copper cations, probably in the forms of CuCl and Cu_2_O.^40^ The peak centered at 933 eV is the confirmation of copper ions bonded with redox-active nitrogen ligands,^41^ as seen also in chalcogenide nanoparticles.^42^

**Figure 5:**
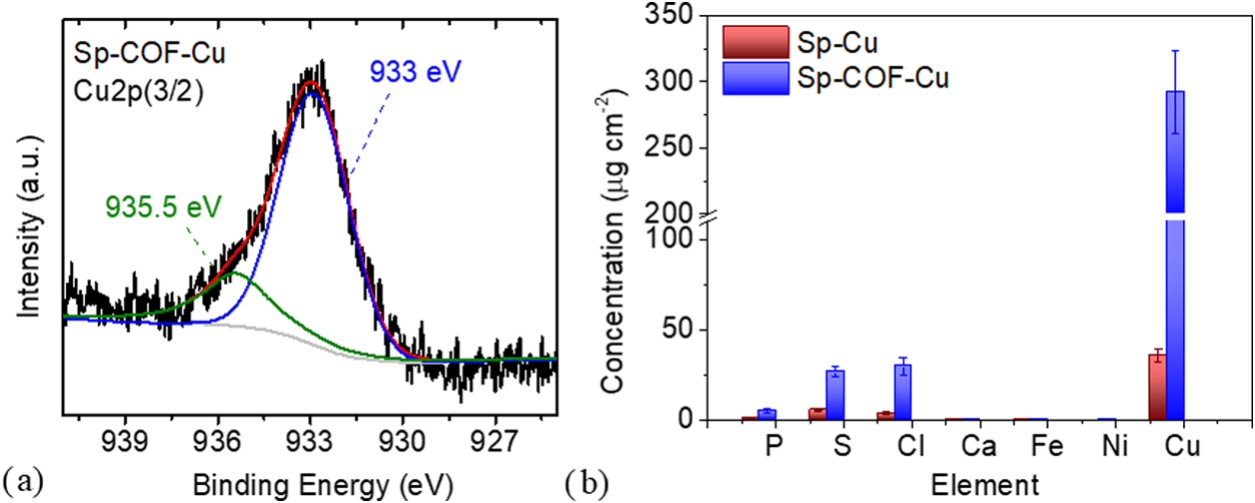
(a) Cu 2p­(3/2) XPS spectra of the Sp-COF-Cu sample demonstrating the presence of strong covalent copper bonds on the surface of the sponge and (b) comparison of the XRF analysis for the Sp-Cu and Sp-COF-Cu samples.

In order to quantify and compare the copper adsorbed by the pristine and modified sponges, XRF measurements under a vacuum were conducted. The ED-XRF spectra of Sp-Cu and Sp-COF-Cu are displayed in Figure S7, and the elemental characterization of the sponges is presented in the comparison chart of Figure 5b, along with the determination of the corresponding concentrations and uncertainty percentages for the traced elements in Table S1. The results demonstrate the superiority of the modified COF sponge over the melamine sponge for Cu adsorption in aqueous environments. The adsorbed quantity was nearly ten times higher on the modified sponge (Sp-COF-Cu) than on the pristine sponge (Sp-Cu). Specifically, the melamine sponge removed 35.9 μg cm^–2^ copper ions, whereas the modified sponge removed 293 μg cm^–2^ from the initial copper sulfate aqueous solution. This large difference is owed to the amount of N pyridine and primary amines that are elevated in the modified sponges and interact directly and strongly with copper ions, in contrast to the secondary amines, for example present in melamine sponges. Studies on the electrostatic potential by Fu et al.^43^ confirm that the active sites for heavy metal absorption are the electron-rich N atoms. Particularly, in pyridine or bipyridine-based covalent organic frameworks, an electron-rich pore from nitrogen atoms is created that greatly facilitates the adsorption of specific metals, such as mercury.^43^

An additional F-AAS experiment for Cu sorption on the COF-modified sponge was conducted to evaluate its % efficiency versus time (min). In Table S2, the corresponding measurements and calculations are gathered, depicting that the sorption starts quickly, even in the first minute. After 25 min, the >90% of a 10 ppm copper sulfate aqueous solution was adsorbed by the Sp-COF-Cu.

### 4.2 Competition between Different Metal Cations

To assess the selectivity of the pristine and modified sponges, an additional adsorption experiment proceeded with the presence of various metal salts, at a 1000 ppm concentration each. The metal salts diluted in water were the following: Ca­(NO_3_)_2_·4H_2_O, Fe­(NO_3_)_3_·9H_2_O, CuSO_4_·5H_2_O, and NiSO_4_·4H_2_O. The photos in Figure S8 depict a short time-lapse of the sponges’ impregnation in the metals’ solutions. The initial solution, where the sponges were immersed for 24 h, has a yellow hue. It is noteworthy to note that once Sp-COF was added to the mixed solution, the modified sponge was instantly colored cyan blue, indicating its selective complexation with the copper. After 24 h, the sponges were collected, washed with deionized water, and left under ambient conditions to evaporate their solvent. In the end, the Sp-metals had a yellow color, while the Sp-COF-metals were colored blue-green.

The FTIR spectra and the XRD patterns of Sp-metals and Sp-COF-metals are demonstrated in Figures S9 and S10, respectively. Similar to the spectra recorded for the copper adsorption (Figure S1), a characteristic −C=N vibrational mode shifting to 1611 cm^–1^ was observed. Other distinguishable peaks are the vibrations of metal-O and metal-N at the low frequency range,^44^ particularly in Sp-COF-metals spectra. At the XRD pattern of the Sp-metals and Sp-COF-metals presented in Figure S10, the reflections of COF, correlated to the 4,4′ bipyridine substituent, and the general in-plane stacking pattern are found at 10.56, 14.6, and 16.02°. Generally, an amorphous pattern is observed in both cases, which supports the absence of an excess of unreacted salts. In Figure S11, the morphology of the sponge-modified frameworks is demonstrated via SEM images. There, the precedence of the amount of the highly adsorbed metals on the modified sponge compared to the pristine sponge is apparent, along with the preservation of the initial microstructure of the commercial sponge. In the EDX mapping and spectrum (Figure S12), a short region of the modified sponge captured, depicts the existence of all of the organic elements and the inorganic compounds.

The Sp-metals sponge is yellow colored (Figure 6a­(i)), while the Sp-COF-metals sponge appears blue-green colored (Figure 6a­(ii)), possibly owing to the adsorption of different metals with a higher quantity of copper. The full survey scan of the XPS measurements of Sp-metals and Sp-COF-metals is included in Figure S13, and the C 1s, N 1s, and Cu 2p­(3/2) peaks are reported in Figure 6b. In that case, identical C 1s spectra were observed, while the O 1s XPS in Figure S14 revealed an additional peak in the lower binding energy at 529 eV, compared to the Sp-COF and Sp-COF-Cu samples, attributed to the creation of metal-oxide.^31,44^ The N 1s region for the Sp-metals has a rather weak signal, attributed to the pyridinic unit of the melamine sponge at 398.6 eV, while for the Sp-COF-metals, there are three intense features in total, similar to the Sp-COF-Cu. The two components of the pyridinic unit from melamine and bipyridine at 399 eV and of phosphazene (type of graphitic N) at 400.5 eV are associated with the COF, and the third one is the new bond created between N and copper ions at 397.7 eV,^45^ as encountered previously. Thus, the adsorption and complexation of copper ions in the presence of other metals are successful and are verified for the Sp-COF-metals. In comparison to the N 1s region of Sp-metals, the signal was rather weak, and there lay only the pyridinic unit of the melamine sponge at 398.6 eV.

**Figure 6:**
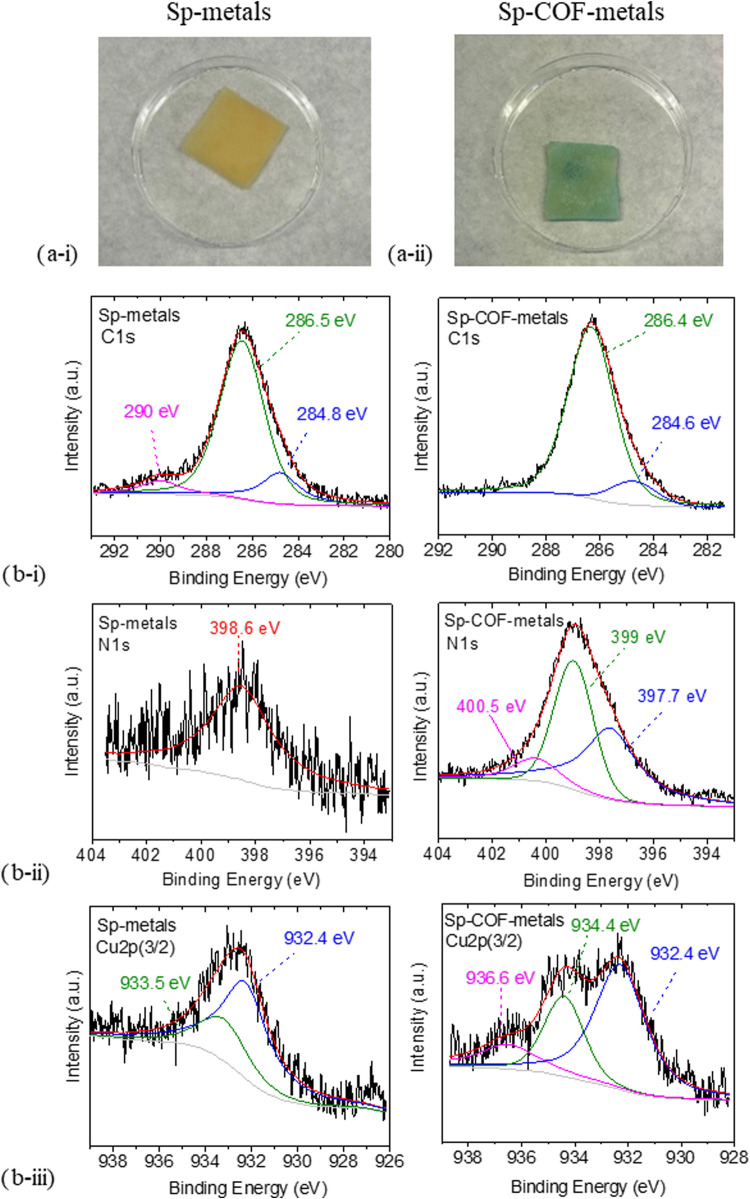
(a) Images of the samples: (i) Sp-metals and (ii) Sp-COF-metals after the adsorption selectivity experiment. (b) XPS measurements of (i) C 1s, (ii) N 1s, and (iii) Cu 2p of the Sp-metals (left) and Sp-COF-metals (right).

In Figure 6b­(iii), the region of Cu 2p­(3/2) of both Sp-metals and Sp-COF-metals is depicted. At the Sp-metals spectrum, two features are fitted at 933.5 and 932.4 eV, indicating clearly a reduction of Cu­(II) to either monovalent or metallic copper, in the presence of the other metal ions. For the Sp-COF metals, the deconvolution of the holistic curve resulted in three components, different from the Sp-COF-Cu. The peak at 932.4 eV is similar to the Sp-metals and signals most probably the presence of monovalent cations.^46^ Thus, different states of copper are considered and cohabitated in the final sample. Generally, XPS cannot distinguish accurately between the Cu^0^ and Cu^+^ species, as their characteristic signals overlap in the same range. However, an XPS signal with a BE of 936.6 eV could be assigned to Cu­(I) coordinated to nitrogen, as found in copper nitrides.^31,46,47^ The peaks of Sp-COF-Cu (935.5 and 933 eV) are not present at Sp-COF-metals, proving that different mechanisms occur during the adsorption of copper ions in the absence and in the presence of other metals.

In Figure S15, the P 2p, Cl 2p, S 2p, Fe 2p, Ni 2p, and Ca 2p regions are presented for further clarification of the mechanisms that occurred in the studied systems. Minor differences are observed between the Sp-metals and the Sp-COF-metals regarding the iron, calcium, and nickel compounds. The nonmetallic components of the COF in Sp-COF- metals, such as P, N, and Cl are identical to the initial Sp-COF. Regarding the P 2p, its peak at 133 eV is attributed to the phosphazene core, akin to the Sp-COF, though it is different from Sp-COF-Cu, which has its peak centered at 135 eV, signaling a lower electron density due to complexation with Cu­(II).^48^ The Cl 2p spectrum at 199.8 eV is centered at the same BE value as the previously mentioned spectrum for the copper modified samples (Figure S5). Displacement reactions and redox reactions should be considered in these complicated systems, since they seem to participate more likely in the adsorption process of the COF-functionalized adsorbent. Along with the absence of nitrate, derivatives from the metal sulfates are no longer present in Sp-COF-metals. In contrast, at the Sp-metals, the binding energy of 168.4 eV is attributed to the SO_4_^2–^ group at the S 2p­(3/2) region.^49^ At the Sp-COF-metals measurements, similar to S 2p, the intensity of Fe 2p is poor, and simulations are not easy to initiate. It is thus highly possible that trivalent iron cations were precipitated as some form of iron hydroxide or oxide. Signals of the Fe 2p­(3/2) region were also weak in Sp-metals, although a curve with a maximum peak near 711 eV is somehow distinguishable, attributed to Fe^3+^ species.^50^ Additionally, the presence of Ni^2+^ in Ni 2p­(3/2) is denoted at 854.7 eV of the Sp-COF-metals spectrum.^51^

In addition to the characterization of the chemical composition, the structure, and morphology of the Sp-metals and Sp-COF-metals, their adsorption capacity in the (1 mg/mL) mixed solution was evaluated by XRF measurements (Figure S16). According to the contributions presented in Figure 7 and Table S3, it is revealed that the concentration afforded Cu was 277 μg cm^–2^, even in the presence of highly concentrated competing metal ions. First, the copper ions are selectively adsorbed among the other metals by the modified sponge until saturation, occupying specific sites, as explained by the EPR study.^23^ Then, the complexation of the 116 μg cm^–2^ Fe and 10.4 μg cm^–2^ Ni transition metals occurs, most likely to other COFs sites, explaining its green-blue color. A small contribution to the adsorption ability could also derive from the non-functionalized areas, attributed to the pristine sponge. For the latter, though, the results showed that only 59.6 μg cm^–2^ of Fe and 0.12 μg cm^–2^ of Cu have been detected, justifying its yellow color. Thus, in the presence of other metals, the modified sponge has 277 times higher performance than the pristine sponge regarding the Cu sorption and two times higher regarding the removal of Fe. In both cases, the metal cations of Ca were not subtracted from the aqueous solution by the sponges.

**Figure 7:**
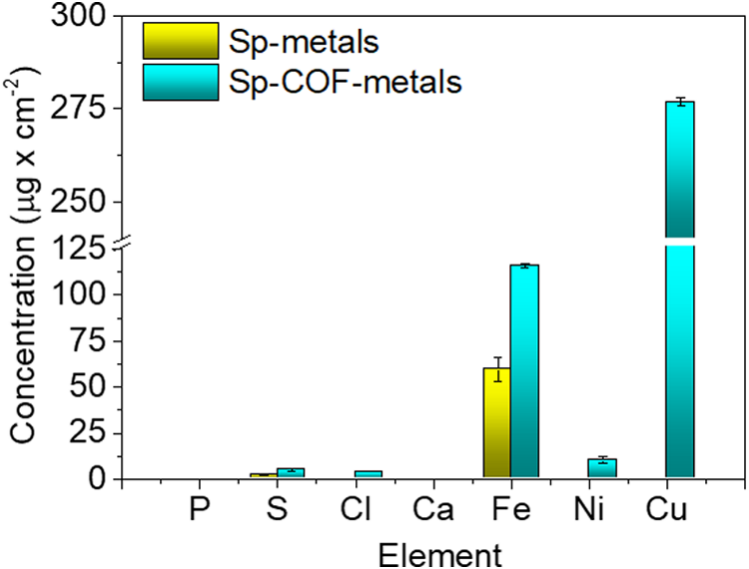
XRF measurements for the elemental contribution in μg cm^–2^ of Sp-metals and Sp-COF-metals.

### 4.3 Recyclability and Electron Paramagnetic Resonance Studies

To commercialize and incorporate the modified sponges in industrial applications, the material needs to be able to desorb and readsorb the targeted metal, while retaining its functionality. The recyclability process can be completed by using a means to detach the copper adsorbed from the Sp-COF-Cu sponge, and afterward, a method to regenerate the COF for another adsorption cycle. The covalent bond developed between the nitrogen of COF with copper is disrupted by an acidic medium; in our case we opted for 2 M HCl, which successfully detached the copper throughout the network. However, our efforts to regenerate and readsorb similar quantities of copper were not promising, probably due to protonation of the functional nitrogen units, leading to a resistance in the formation of other states or complexes of copper. Deprotonation procedures^52^ were attempted only to create, after the readsorption, a few other copper compounds and a slight traceable amount of divalent copper by EPR, as explained ahead.

Electron Paramagnetic Resonance spectroscopy is a powerful tool for the detection and identification of divalent copper cations and their chemical environment. At Q-band, the resolution of the *g* values of the different Cu^2+^ species is optimized due to the higher frequency (34 GHz) compared to X-band.^53^ After recording the Q-band EPR spectra of the Sp-Cu, Sp-COF-Cu, Sp-metals, and Sp-COF-metals, their approximation by fitting was completed, and the simulation parameters are presented in Table S4. To that end, the Q-band EPR spectra of the Sp-Cu and Sp-COF-Cu are presented in Figure 8a. The spectrum of the Sp-COF-Cu was approximated by fitting with four major species of the following *g* values and percentages: (*g*1, *g*2, *g*3, %) = (2.052, 2.092, 2.35, 31.7), (2.076, 2.099, 2.291, 29.3), (2.035, 2.154, 2.198, 22.7), (2.079, 2.247, 2.259, 14.1). The first two species show square planar geometry, while the other two are typical of trigonal bipyramidal geometry. The spectrum of the Sp-Cu alone seems to be represented mostly by the trigonal bipyramidal species with *g* values of (2.079, 2.247, 2.259). It can be assumed that this kind of trigonal bipyramidal Cu is bound only to the melamine sponge part and not the part of the COF for both samples, while the rest of the species are more associated with their covalent bond on the COF. Unfortunately, as explained in the previous paragraph, the second cycle of the reabsorption experiments on the Sp-COF did not result in an appreciable amount of divalent copper to offer an intense EPR signal (not shown). After the adsorption experiment in the mixed solution of the competitive metal salts, the spectrum of the Sp-COF-metals (Figure 8b) was approximated by fitting with 3 species with the following *g* values and percentages: (*g*1, *g*2, *g*3, %) = (2.032, 2.149, 2.189, 50), (2.032, 2.141, 2.201, 42), (2.047, 2.089, 2.152, 8). It is noted that in the presence of other metals, the binding of copper to COF was coordinated particularly in a trigonal bipyramidal geometry (represented by the great majority). In contrast, the Sp-metals sample exhibited a very weak EPR spectrum, indicating almost no Cu adsorption in the presence of the other metal ions (not shown). However, the XPS study verified the adsorption of a small percentage, attributed to either the metallic or the monovalent copper. These oxidation states of copper cannot be traced through EPR, as only Cu^2+^ is EPR active. The different species verified from EPR in Sp-COF-Cu and Sp-COF-metals are in agreement with the results obtained by the XPS study. There are different states of copper influencing the signals, as well as different groups bonded to copper. Even though at the Sp-COF-Cu, trigonal bipyramidal geometries, similar to those reported in our previous work,^23^ along with the square planar geometry, are developed; the competitive metal ions in Sp-COF-metals are not permitting the copper ions to balance in positions other than their preferential trigonal bipyramidal. In summary, (i) the divalent copper in Sp-COF-Cu is coordinated in both square planar and trigonal bipyramidal, represented by two species each with different substituents, one also exists in Sp-Cu and (ii) the Sp-COF-metals sample exhibits exclusively the trigonal bipyramidal coordination, while the Sp-metals demonstrates no distinctive EPR signals of the divalent copper ions.

**Figure 8:**
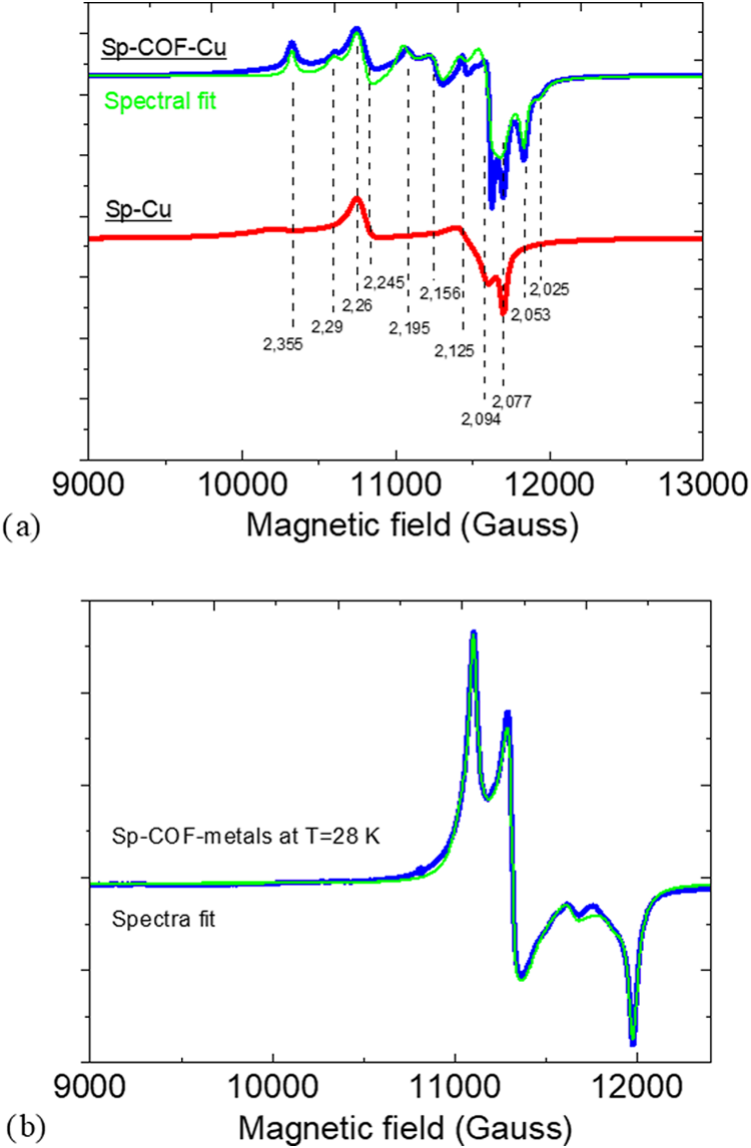
(a) Q-band EPR spectra recorded at room temperature for the Sp-Cu and Sp-COF-Cu alongside the fitting curve and a spectrum recorded after twisting the tube at 90°. (b) Q-band EPR recorded at *T* = 28 K for the Sp-COF-metals.

## 5 Conclusions

A phosphazene-based covalent organic framework was successfully functionalized on commercial melamine sponges, and the advanced modified system was applied for the removal of copper cations from aqueous solutions. We revealed that the attachment of the COFs on the sponge is a necessity for the visual detection and the efficient adsorption of copper in comparison to pristine sponges. XPS analysis demonstrated the creation of a covalent bond between the COF and the copper ions, while at the same time, it was concluded that a partial reduction of the divalent copper may take place in the presence of competitive metal cations. By means of EPR spectroscopy, the presence of divalent copper in different functional sites and geometries was identified, depending on the measured sponge, pristine or modified, and on the cohabitation of other metal salts in the initial solutions. This work presents fundamental importance in the field of water management and remediation as it paves the way for applications of the selective adsorption for other toxic heavy metals, such as lead and mercury, by post-modification of the frameworks in low-cost, commercial scaffolds. Finally, another key aspect of our forthcoming efforts will be the improvement in the recyclability of the adsorber.

## Supplementary Material



## Notes

Additional FTIR spectra; XRD patterns; XPS spectra alongside their fitting curves; XRF spectra; images of the solutions and the sponges; SEM and EDX images; Table of the EPR simulation parameters (PDF)
