# Supplementary material for: Detection and Selective Sorption of Copper Ions by a COF-Modified Melamine Sponge
Source: ACS Omega. 2025 May 21;10(21):21755–66. doi: 10.1021/acsomega.5c01393 (PMC12138691; doi:10.1021/acsomega.5c01393)
Supplement: Supplementary file 1 [file ao5c01393_si_001.pdf]

Detection and selective sorption of copper ions by a COF modified melamine sponge

Panagiota Bika [a], Nikolaos Ioannidis [a], Polychronis Tsipas [a, c], Stefanos Papagiannis [b], Maria-Anna Gatou [d], Evangelia A. Pavlatou [d], Andreas Germanos Karydas [b], Thomas Stergiopoulos [a] and Panagiotis Dallas [a, e, *]

[a] *Institute of Nanoscience and Nanotechnology, NCSR Demokritos, 15341, Athens, Greece*

[b] *Institute of Nuclear and Particle Physics, NCSR Demokritos, 15341, Athens, Greece*

[c] *National Institute of Materials Physics, Atomistilor 405A, Magurele, Romania*

[d] *Laboratory of General Chemistry, School of Chemical Engineering, National Technical University of Athens, Zografou Campus, 15772, Athens, Greece*

[e] *Theoretical and Physical Chemistry Institute, National Hellenic Research Foundation, 11635, Athens, Greece*

- p.dallas@inn.demokritos.gr; pdallas@eie.gr

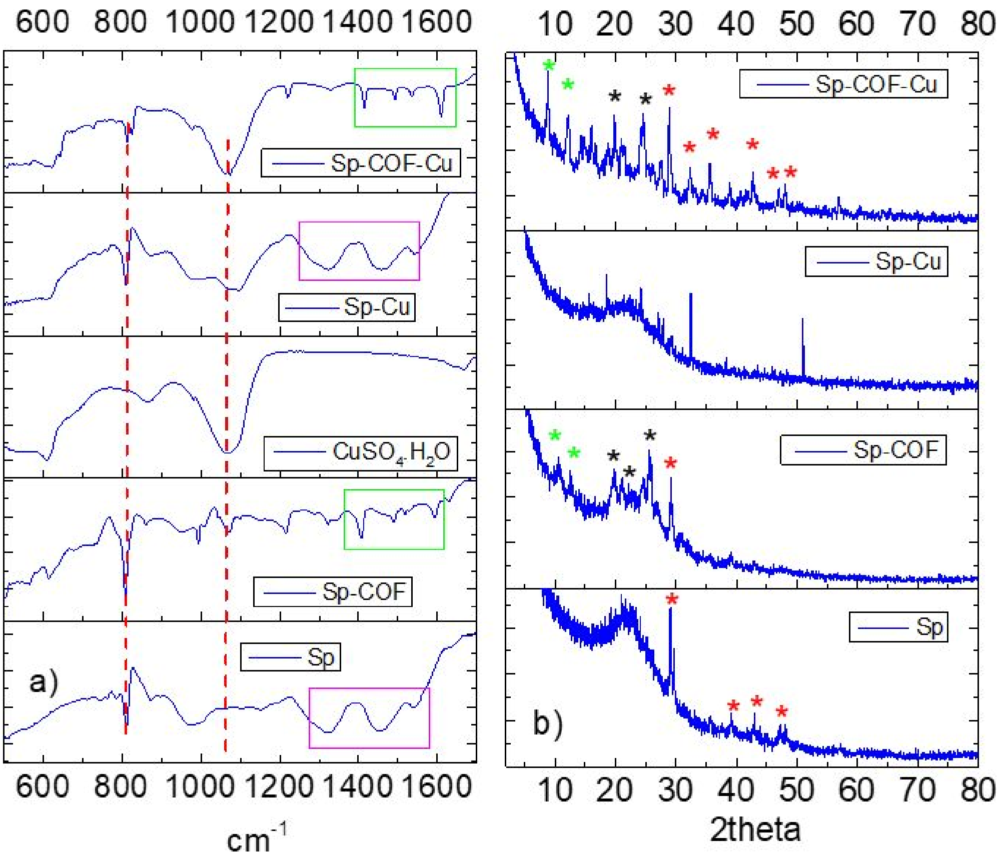

Figure S1. a) The FTIR spectra and b) the XRD patterns of the Sp, Sp-COF, Sp-metals and Sp-COF-metals samples. Red stars: holder and tack.

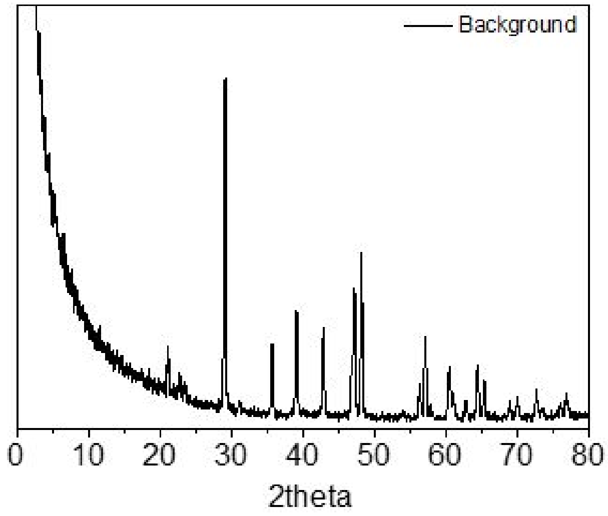

Figure S2. XRD patterns of the background holder and the 'tack' for the samples' support during the measurement.

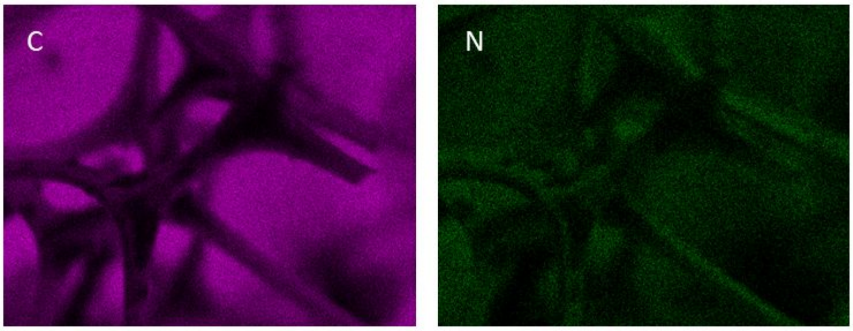

Figure S3. The EDX mapping of the C and N elements of the selected region in Sp-COF-Cu presented in Figure 3.

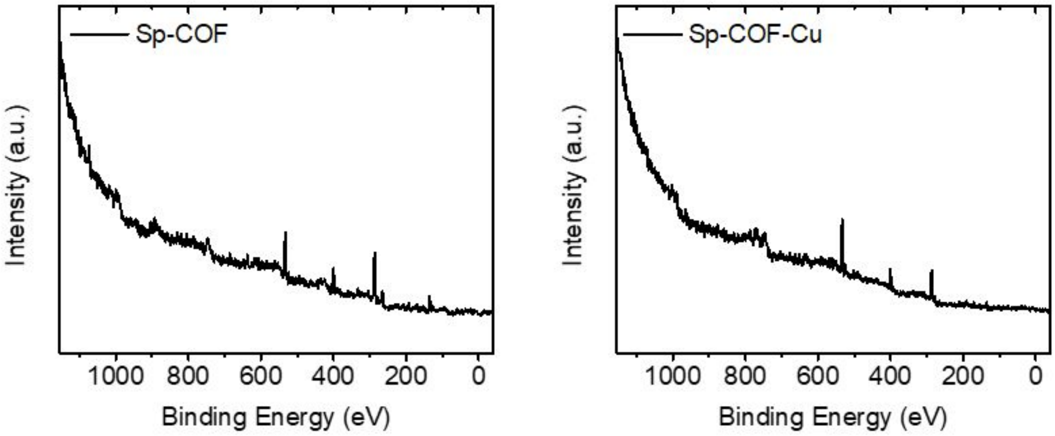

Figure S4. The XPS full survey scan of the Sp-COF (left) and Sp-COF-Cu (right) spectra**.**

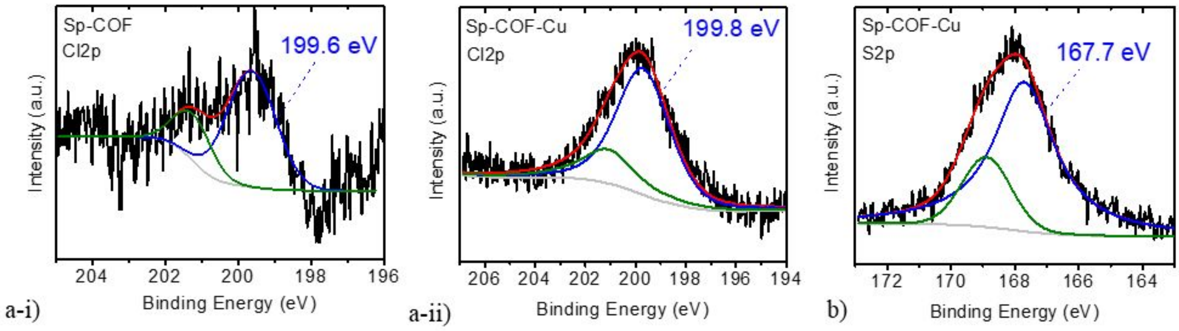

Figure S5. a) Cl2p XPS spectrum of Sp-COF (i) and Sp-COF-Cu (ii) and b) S2p XPS spectrum of the Sp-COF-Cu.

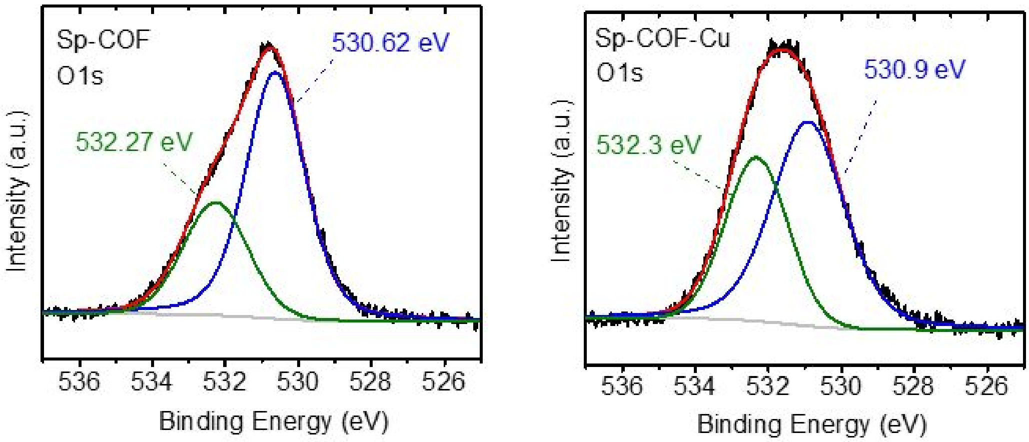

Figure S6. O1s XPS of the Sp-COF (left) and Sp-COF-Cu (right) samples.

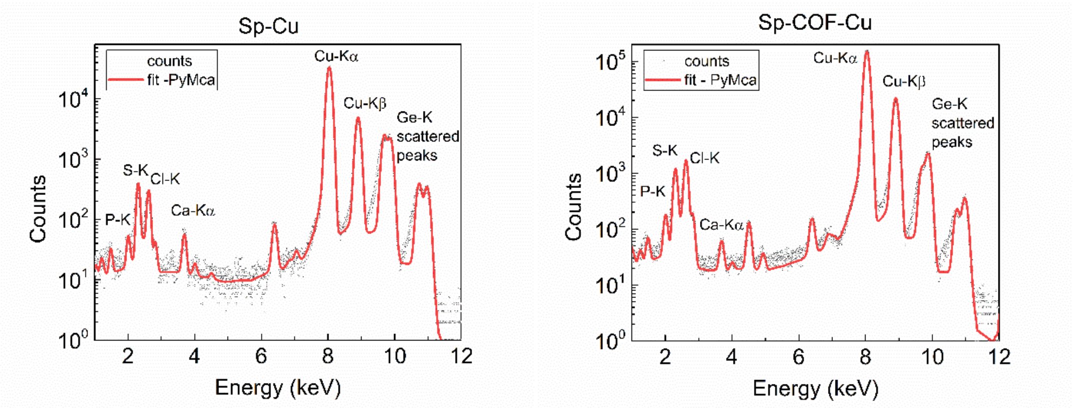

Figure S7. XRF spectra of Sp-Cu (left) and Sp-COF-Cu (right) samples, along with the detected elements.

Table S1. XRF results for the Sp-Cu and Sp-COF-Cu.

**Element**	**Concentration μg cm^{-2}**	**Uncertainty (%)**	**Concentration μg cm^{-2}**	**Uncertainty (%)**
	**Sp-Cu**		**Sp-COF-Cu**	
**P**	0.76	26.5	5.1	26.5
**S**	5.4	9.3	26.8	9.3
**Cl**	3.7	16.6	29.6	16.6
**Ca**	0.19	11.2	0.32	11.0
**Cu**	35.9	10.7	293	10.7

Table S2. F-AAS results for the adsorption of Cu^{2+} by Sp-COF-Cu: Cu precursor: $CuSO_4$ nH_2O (Cu^{2+}), initial Cu $^{2+}$ concentration (C_0) measured by F-AAS 9.44 ppm,

flow speed: 2 rpm (0.35 mL min-1), V (initial solution): 250 mL and sorbent mass (m. in g): 0.3

**t (min)**	**Column a**	**Column b**	**Column c**	**SDEV**	**Average C_t value (ppm)**	**C_0-C_t**	**(C_0-C_t)/C_0**	**Removal (%)**	**Sorbed metal concentration (q, mg/g)**
	C_{Cu} (ppm)	C_{Cu} (ppm)	C_{Cu} (ppm)						
**1**	9.19	9.23	9.15	0.04	9.19	0.25	0.0265	2.65	0.02
**5**	7.44	7.22	7.31	0.11	7.32	2.12	0.2242	22.42	0.18
**10**	6.89	6.67	6.83	0.11	6.80	2.64	0.2800	28.00	0.22
**15**	3.55	3.87	3.68	0.16	3.70	5.74	0.6081	60.81	0.48
**20**	2.23	2.17	2.39	0.11	2.26	7.18	0.7602	76.02	0.60
**25**	0.67	0.72	0.69	0.03	0.69	8.75	0.9266	92.66	0.73
**30**	0.57	0.55	0.61	0.03	0.58	8.86	0.9389	93.89	0.74
**35**	0.34	0.45	0.38	0.06	0.39	9.05	0.9587	95.87	0.75
**40**	0.28	0.27	0.33	0.03	0.29	9.15	0.9689	96.89	0.76
**45**	0.19	0.15	0.16	0.02	0.17	9.27	0.9823	98.23	0.77
**50**	0.18	0.14	0.18	0.02	0.17	9.27	0.9823	98.23	0.77
**55**	0.12	0.09	0.09	0.02	0.10	9.34	0.9894	98.94	0.78
**60**	0.03	0.02	0.06	0.02	0.04	9.40	0.9961	**99.61**	0.78

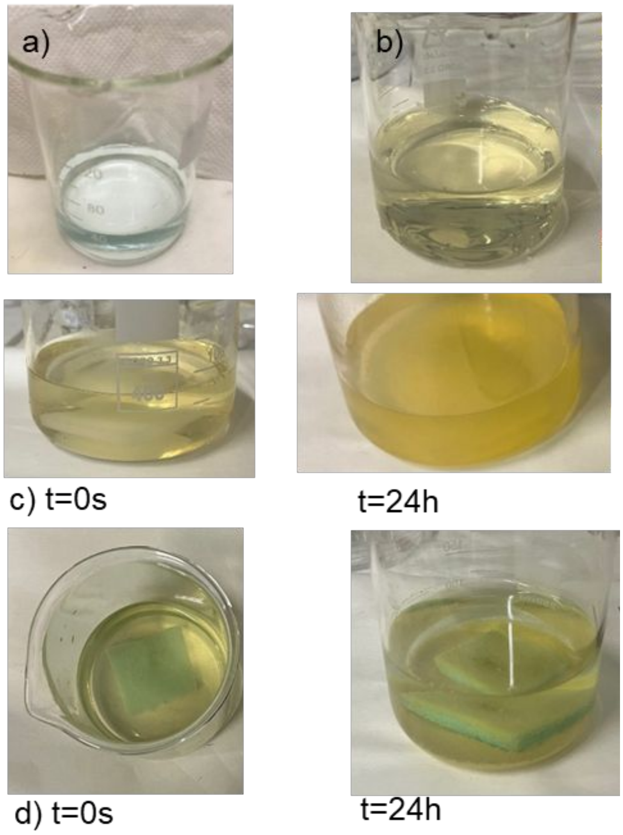

Figure S8. a) Stock solution of copper sulfate b) Stock solution of calcium nitrate, iron nitrate, copper sulfate, nickel sulfate, c) Sp d) Sp-COF, at t=0 s, when the impregnation started and d) at the end of the adsorption experiment (t=24 h).

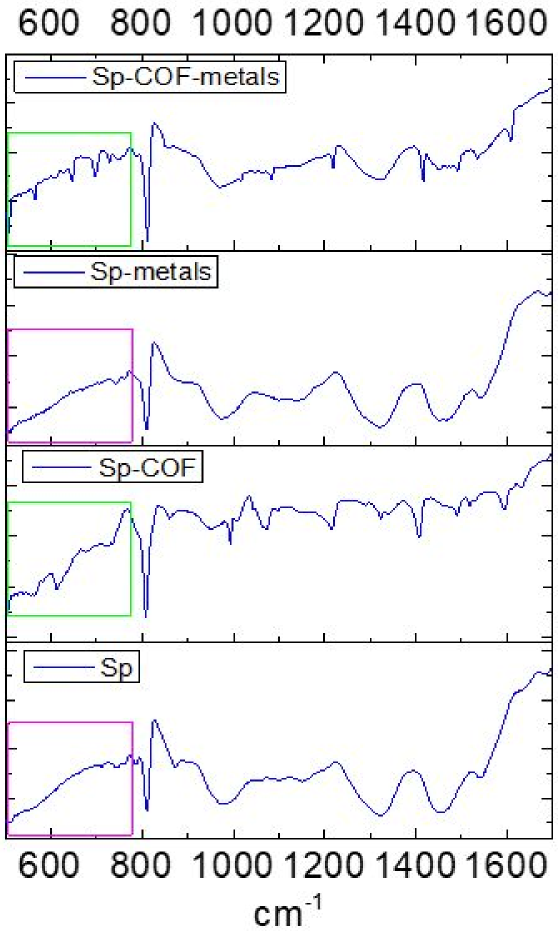

Figure S9. FTIR spectra of the Sp; Sp-COF; Sp-metals and Sp-COF-metals samples.

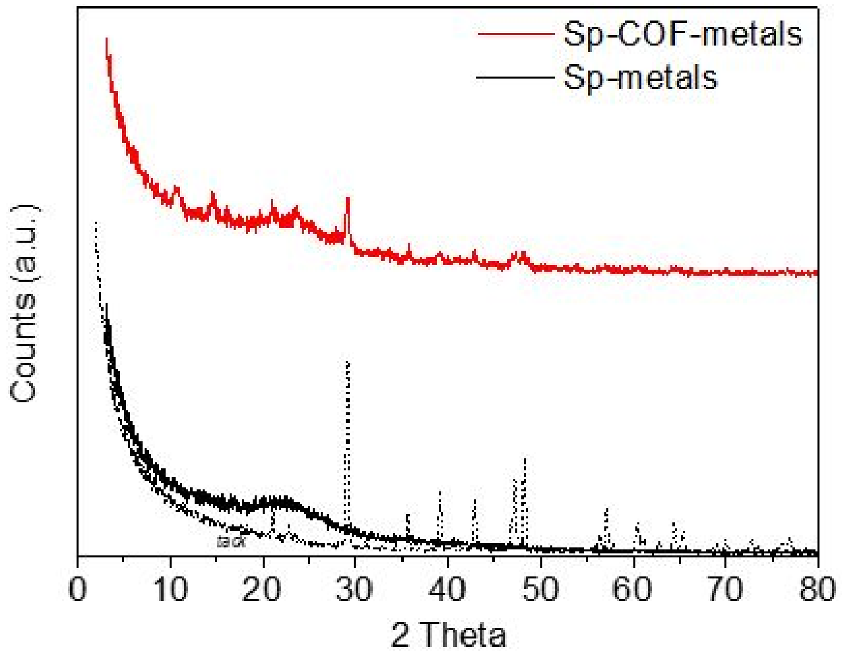

Figure S10. XRD patterns of Sp-metals and Sp-COF-metals, along with the ‘tack’ for the samples’ support during the measurement.

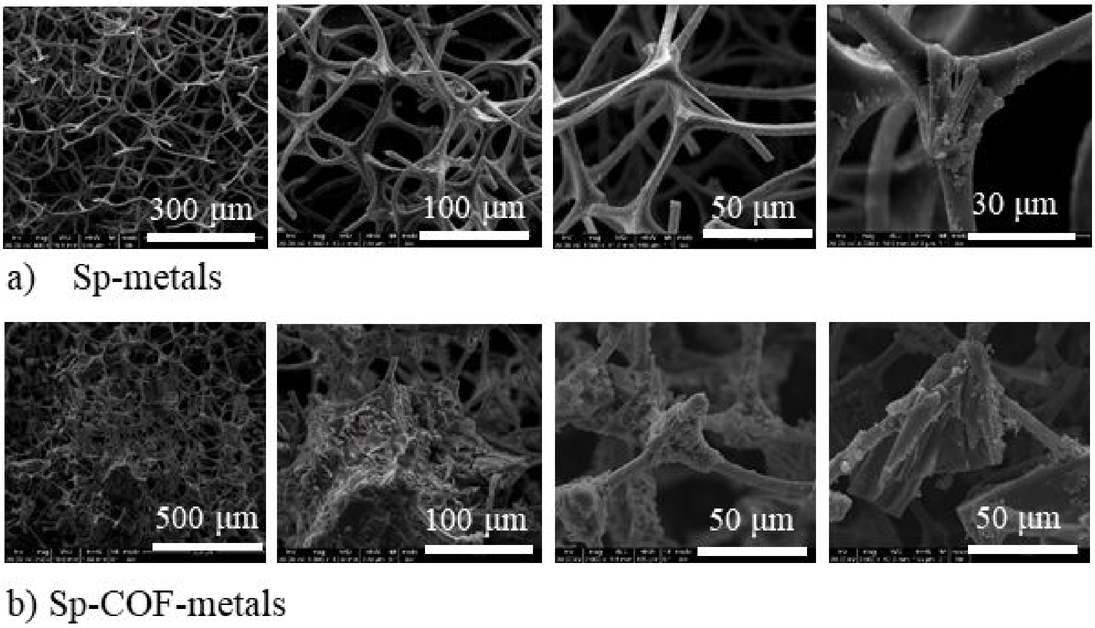

Figure S11. SEM images of a) Sp-metals and b) Sp-COF-metals samples.

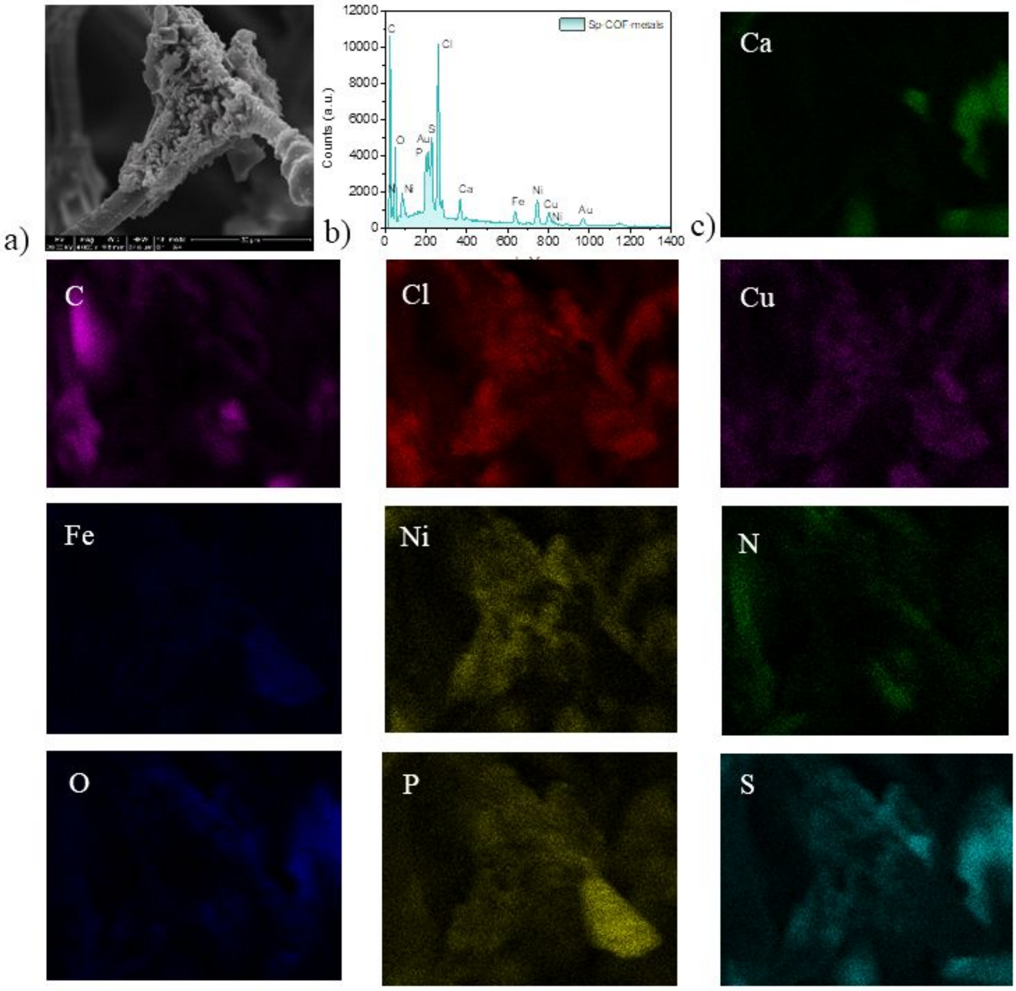

Figure S12. a) SEM picture of a representative region of the Sp-COF-metals sample, b) the corresponding EDX spectrum and c) the EDX mapping of the C, P, N, Cl, Cu, Fe, Ni, Ca, S elements.

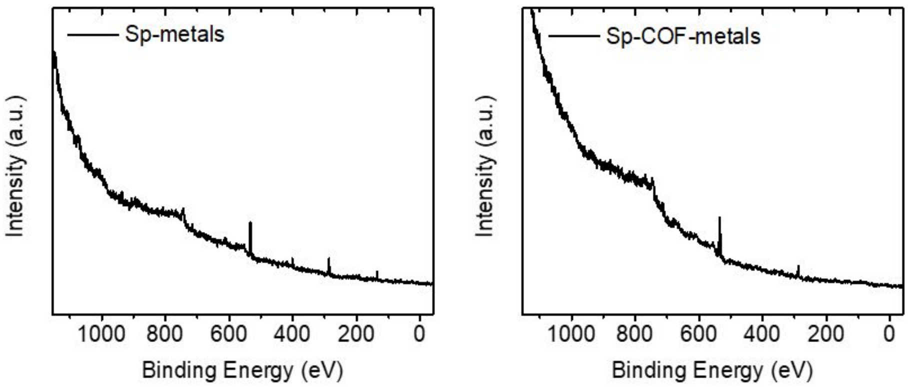

Figure S13. The XPS full survey scan of the Sp-metals (left) and Sp-COF-metals (right) spectra

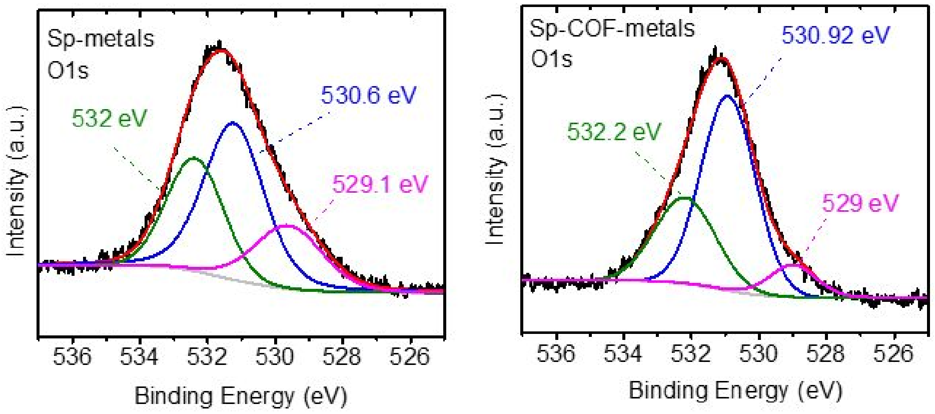

Figure S14. O1s spectra for the Sp-metals (left) and Sp-COF-metals (right).

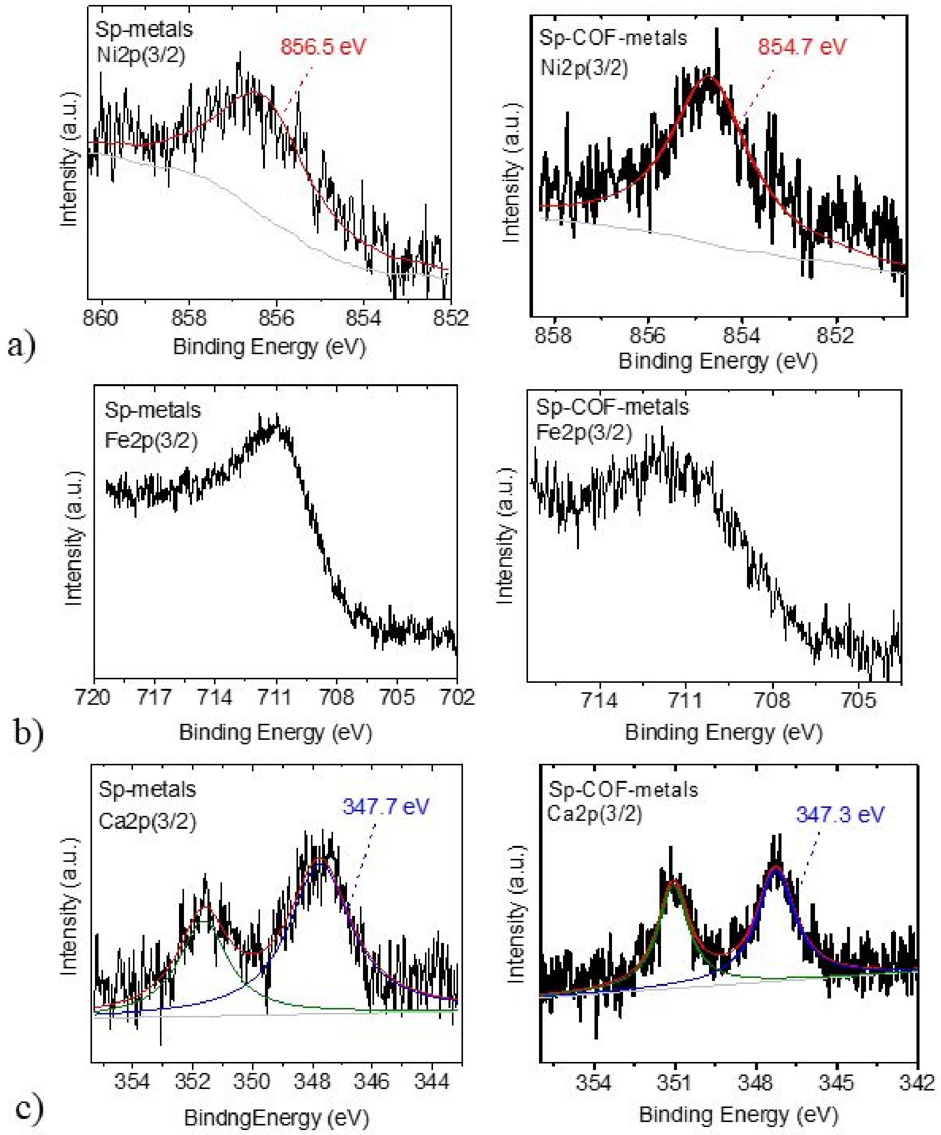

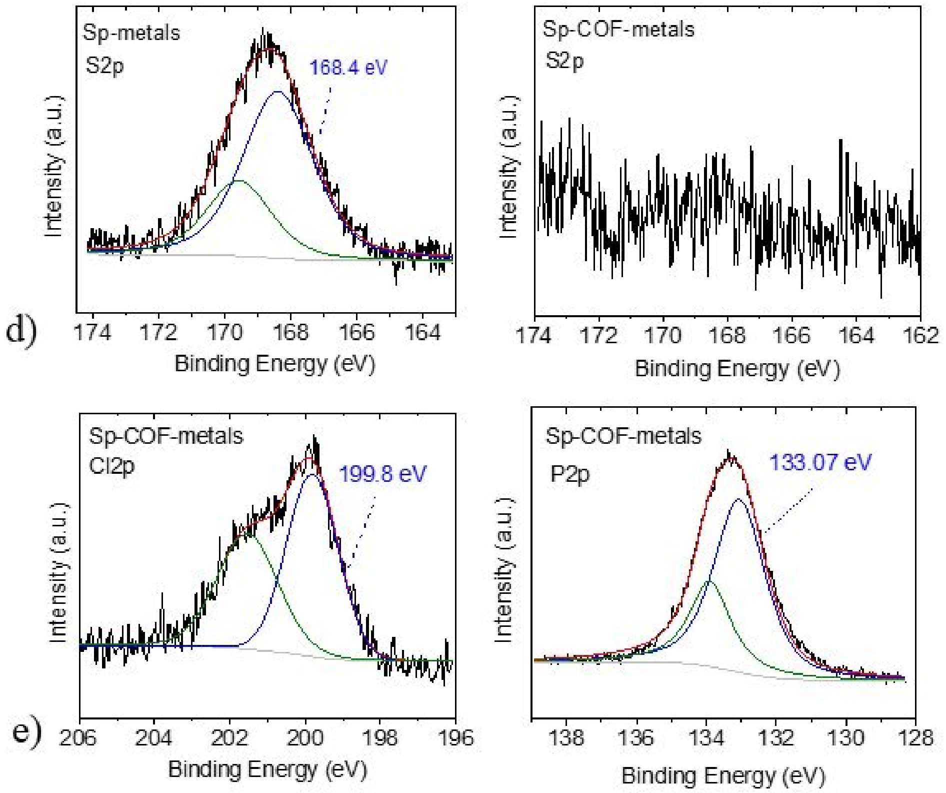

Figure S15. XPS spectra of (a) iron, (b) nickel, (c) calcium, and (d) sulfur elements for the samples: Sp-metals (left), Sp-COF-metals (right) and e) the Cl2p and P2p spectra for the Sp-COF-metals.

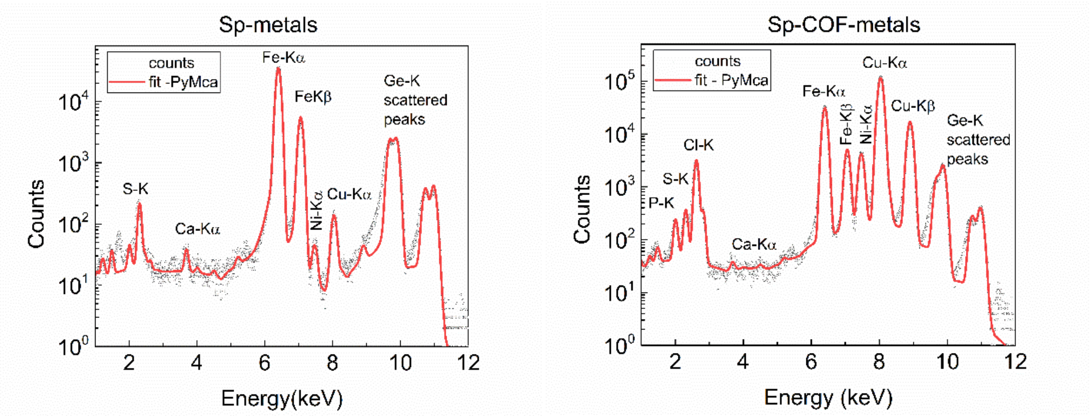

Figure S16. XRF spectra of Sp-metals (left) and Sp-COF-metals (right) samples, along with the detected elements.

Table S3. XRF results for the Sp-metals and Sp-COF-metals.

Element	Concentration $\mu g\ cm^{-2}$	Uncertainty (%)	Concentration $\mu g\ cm^{-2}$	Uncertainty (%)
	Sp-metals		Sp-COF-metals	
**P**				
**S**	2.4	9.3	5.0	9.3
**Cl**			3.4	16.6
**Ca**				
**Fe**	59.6	10.7	116	10.7
**Ni**	0.036	13.5	10.4	13.1
**Cu**	0.12	10.8	277	10.7

Table S4. The EPR simulation parameters of the samples Sp-Cu, Sp-COF-Cu, Sp-metals and Sp-COF-metals.

Sample	g1	g2	g3	%	Geometry
**Sp-Cu**	2.079	2.247	2.259	~100	TB
**Sp-COF-Cu**	2.052	2.092	2.35	31.7	SP
	2.076	2.099	2.291	29.3	SP
	2.035	2.154	2.198	22.7	TB
	2.079	2.247	2.259	14.1	TB
**Sp-COF-metals**	2.032	2.149	2.189	50	TB
	2.032	2.141	2.201	42	TB
	2.047	2.089	2.152	8	Intermediate of TB and SP
